# Agreement of Sleep Measures—A Comparison between a Sleep Diary and Three Consumer Wearable Devices

**DOI:** 10.3390/s22166189

**Published:** 2022-08-18

**Authors:** Kristina Klier, Matthias Wagner

**Affiliations:** Fakultät für Humanwissenschaften, Institut für Sportwissenschaft, Universität der Bundeswehr München, 85577 Neubiberg, Germany

**Keywords:** concordance, sleep, sleep assessment, sleep diaries, self-tracking, wearable devices

## Abstract

Nowadays, self-tracking and optimization are widely spread. As sleep is essential for well-being, health, and peak performance, the number of available consumer technologies to assess individual sleep behavior is increasing rapidly. However, little is known about the consumer wearables’ usability and reliability for sleep tracking. Therefore, the aim of the present study was to compare the sleep measures of wearable devices with a standardized sleep diary in young healthy adults in free-living conditions. We tracked night sleep from 30 participants (19 females, 11 males; 24.3 ± 4.2 years old). Each wore three wearables and simultaneously assessed individual sleep patterns for four consecutive nights. Wearables and diaries correlated substantially regarding time in bed (Range CCC_Lin_: 0.74–0.84) and total sleep time (Range CCC_Lin_: 0.76–0.85). There was no sufficient agreement regarding the measures of sleep efficiency (Range CCC_Lin_: 0.05–0.34) and sleep interruptions (Range CCC_Lin_: −0.02–0.10). Finally, these results show wearables to be an easy-to-handle, time- and cost-efficient alternative to tracking sleep in healthy populations. Future research should develop and empirically test the usability of such consumer sleep technologies.

## 1. Introduction

The important role of high sleep quality for well-being, health, and peak performance is well acknowledged [1,2,3]. In terms of health literacy, proactive and targeted dealing with own sleep patterns is also becoming increasingly relevant [4,5]. A popular trend in this context is self-tracking by using wearable consumer sleep technologies such as fitness trackers or smartwatches as easy to handle and time- and cost-efficient tools [6,7,8]. Wearables are portable sensors that track activity combined with the recording of physiological parameters such as heart rate or body temperature. Connected to a mobile application, this information is usually available to the consumer in graphic form on an ad hoc basis. However, the measurement accuracy of respective devices and the requirements for handling the collected data still need to be seen as critical [9,10,11,12]. Hence, there is a need for research investigating the measurement accuracy, especially of market-leading devices, in order to prove the validity and reliability of their outcomes [13]. As many people already use such wearables to track their everyday activities, the additional recording of sleep quality using the same system is cost-efficient and highly feasible [14,15]. It might be considered that wearables measure what they claim to measure, but at the same time, due to the technical development of the devices and the underlying algorithms, which are constantly adapted, it can be assumed that they generally do not come 100% close to the values of the gold standard or even do not assess all parameters measurable in polysomnography (PSG; [16,17,18,19,20,21]). It should be noted that lacking access to the raw data of wearables does not allow any quantification or overall associated statistical comparison of sleep stages. Researchers are currently approaching this problem by developing their own algorithms or programming neural networks to capture raw data [22,23,24,25]. This, in turn, reveals the need for research regarding the feasibility and practical implications for the devices’ usability [26,27,28]. Accordingly, as most of the sleep research has been conducted in the laboratory setting and little is known about the consumer wearable devices’ ability to measure sleep in free-living conditions, the aim of the present study was to assess the level of agreement of sleep measures between a standardized sleep diary, i.e., the gold standard in non-laboratory conditions, and three available consumer sleep technologies.

## 2. Materials and Methods

### 2.1. Study Conception and Procedure

**Figure 1:**
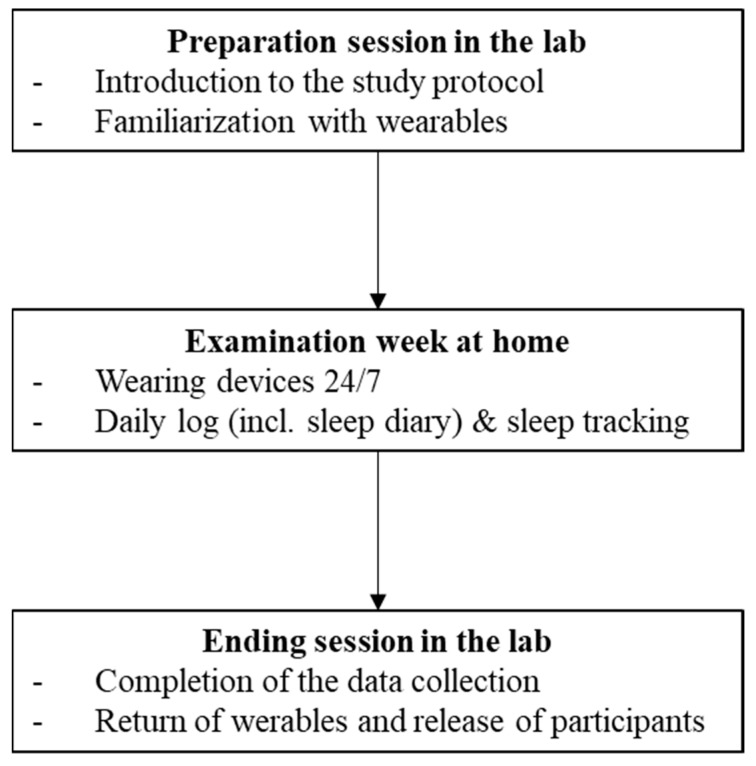
Flow Chart of the examined study protocol.

To compare the sleep measures from the sleep diary and three common consumer wearable devices, participants were asked to assess their sleep for four consecutive nights. The study was designed as a within-subject protocol and lasted ten weeks overall. Figure 1 illustrates the examined study protocol.

The investigations started on Mondays with the collection of relevant anthropometric data and an introduction to the assessment procedure. In addition to creating customer accounts, the latter included setting up the devices individually, explaining how to fill in the daily log (including the sleep diary), and finally clarifying open questions. The wearables should always be worn on the non-dominant wrist. Their arrangement was randomly assigned. From Tuesday to Friday, participants kept their daily routine, during which they wore the wearables with as few interruptions as possible. Every morning after filling in the log, participants sent the tracked sleep data as screenshots from the watch-related apps/platforms to the study administration. After these four consecutive nights, the investigation ended on Fridays with the return of the wearables and reception of individual feedback on handling the devices as well as on the subjective rating of the wearables and their measurements. Finally, the wearables were reset and the accounts deleted in order to prepare them for the following subjects.

### 2.2. Participants

Thirty participants (mean age = 24.3 years (SD 4.3; Range 19–35), mean BMI = 23.3 (SD 1.9; Range 20.3–29.3), 11 males, 19 females) were recruited via students’ and employees’ mailing lists in the authors’ institutional context. The university’s ethics committee approved all procedures, and all participants provided informed written consent. The research was conducted in accordance with the Declaration of Helsinki [29]. As participation was voluntary and did not involve any further risks, healthy young adults who did not suffer from diagnosed sleep disturbances, were injured, ill, or pregnant were included in the study. Based on the medium effect size reported in Lee et al. [30], a priori G*Power analysis [31] predicted a required sample size of N = 26 (*p* = 0.05, d = 0.5, 1 − β = 0.80).

### 2.3. Materials

Three different wearables were used for the objective measurement of sleep. We chose wearables from Garmin^®^, Polar^®^, and Fitbit^®^ as these are well-known brands, often used, and recommended (see, for example, [9,32]). All three devices measure movement using a 3D accelerometer and heart rate based on photoplethysmography. Technical specifications of the individual devices can be found in Table 1.

*Versa^®^* 2 is a wrist smartwatch from Fitbit^®^ Inc., San Francisco, CA, USA (model year 2019) that is located in the middle price segment and is designed for holistic use in daily life. In addition to the classic wristwatch functions, it is mainly characterized by the GPS function and 24-h heart rate monitoring. Sleep and relaxation modules as well as other available apps broaden the functioning spectrum. Hence, the compatibility with IOS and Android supports these usage options. *Fēnix^®^ 5X Plus* is a wrist sports watch from Garmin^®^ Ltd., Olathe, KS, USA (model year 2018). As a GPS multisport watch, it is one of the high-end products (middle-to-upper price segment) of the current market, which can be used both in everyday life and specifically as a training watch. According to the compatibility with IOS and Android, there are a large number of overarching usage functions. *Ignite^®^* is a wrist sports watch from Polar Electro^®^ Oy. Kempele, Finland (model year 2019). As a fitness watch in the middle price segment, it is primarily designed for analyzing and controlling physical and sporting activities. Numerous training modes, the recording and monitoring of several vital parameters, as well as the compatibility with IOS and Android, enable the watch to be used in various ways.

As it is not only important to observe quantitative parameters for a comprehensive understanding of sleep, the combination of objective and subjective measurement methods is recommended. Therefore, the standardized evening-morning protocol [33], which is a valid tool in sleep medicine, was included for the subjective assessment of sleep. Immediately before going to bed and after waking up, six or rather eight questions on the state of mood and sleep quality needed to be answered. Usually (i.e., mainly in a clinical setting), this daily logging covers a period of two weeks. An individual period of time can be implemented for healthy subjects or to estimate a tendency. For evaluation, the means of time in bed, sleep onset latency, sleep duration, and waking frequency and duration were taken. Mood and tiredness in the morning as well as in the evening were also averaged. However, for interpretation, the calculation of sleep efficiency is more decisive. Hereby, values between 80 and 90% are considered normal, although a high subjective sleep efficiency does not necessarily imply a good objective sleep quality.

### 2.4. Data Analysis

We used the data processing programs Excel (Microsoft, 2019) for data preparation and SPSS Statistics Version 27 (IBM, Inc., Chicago, IL, USA, 2021) for the statistical analysis. The level of significance was set a priori at α = 0.05. To analyze the wearables’ usability, we performed descriptive statistics and conducted an examination of the devices’ success or failure. For proving the reliability, we first created Bland-Altman plots (B-A-P; [34]) for graphic interpretation and the detection of outliers. Second, we calculated the Lins concordance coefficient (CCC_Lin_; [35]). Third, to answer the question of whether mean value differences scatter systematically over the range of the x-axis, we verified the assumption of normality of the data using the Shapiro-Wilk test (S-W-T; [36]) and assessed the assumption of homoscedasticity using the modified Breusch–Pagan test (B-P-T; [37]). If data were not normally or heteroscedastic distributed, we performed a logarithmic transformation. For a better interpretation of the resulting Limits of Agreements of the original measurements, we performed an inverse transformation using the ‘antilog’ function [38] and, finally, computed the percentage deviation of mean value differences. It should be noted that, in the statistical analysis, only those sleep parameters were included that were available respectively calculable for the sleep diary and all three wearables: time in bed (TIB), total sleep time (TST), sleep efficiency (SE), sleep stages (SS), and sleep interruptions (WASO). All variables were calculated in minutes, except SE which is expressed as a percentage. Unless otherwise stated, all data are given as means and standard deviations ($\bar{x}$ ± SD).

## 3. Results

The following results section is split in line with the two main analyses we have performed. To gain first insights, we first present the descriptively analyzed sleep measurements. We then focus on the comparison of the wearables and the sleep diary. This agreement section is partitioned according to the considered sleep parameters.

### 3.1. Descriptives

In total, 120 nights were recorded with an overall failure rate of 4.1% (Fitbit^®^ 9.8%, Garmin^®^ 0%, Polar^®^ 1.7%). Either hardware/software errors or human errors could have influenced the outcome. For example, whereas data loss was caused by the software’s inability to detect any data or the device’s inability to connect with the software and download data, participants might have failed to use the devices’ “sleep mode” or correctly document and save the nightly recordings. In line with the examined missing sleep values, participants rated the handling and form of data presentation/availability poorest for Fitbit^®^, whereas Polar^®^ and Garmin^®^ ranked equally best.

Participants’ sleep characteristics are presented in Table 2. Although participants slept while wearing multiple devices on one arm, mean values of sleep variables were in the normal range of prevailing sleep guidelines [39].

**Figure 2:**
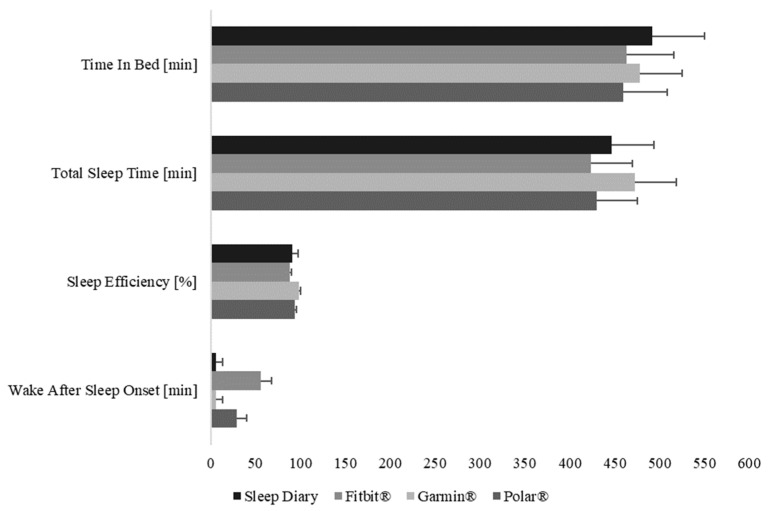
Overview of the analyzed sleep variables presenting time parameters in minutes and sleep efficiency in percentage.

A first graphical comparison between the devices in the further inferential statistical analysis considering sleep variables is shown in Figure 2.

### 3.2. Agreement between Wearables and Sleep Diary

**Figure 3:**
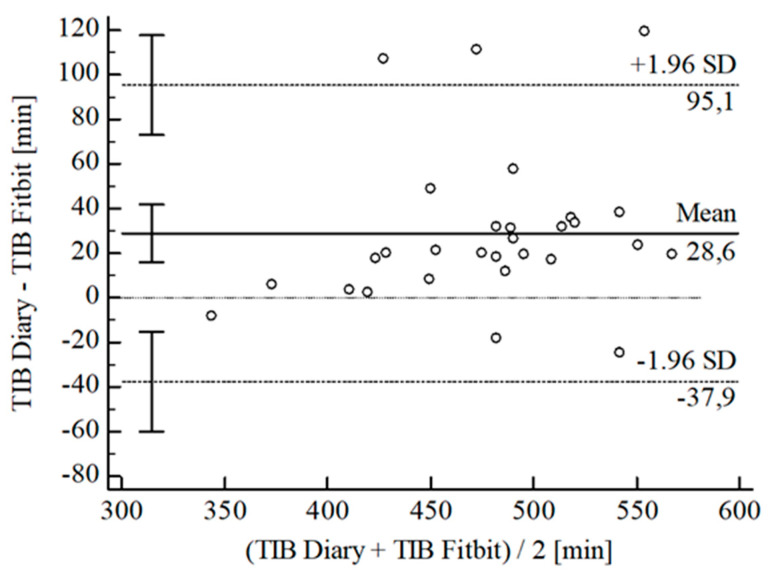
Bland-Altman plot for TIB Fitbit^®^ vs. diary.

**Figure 4:**
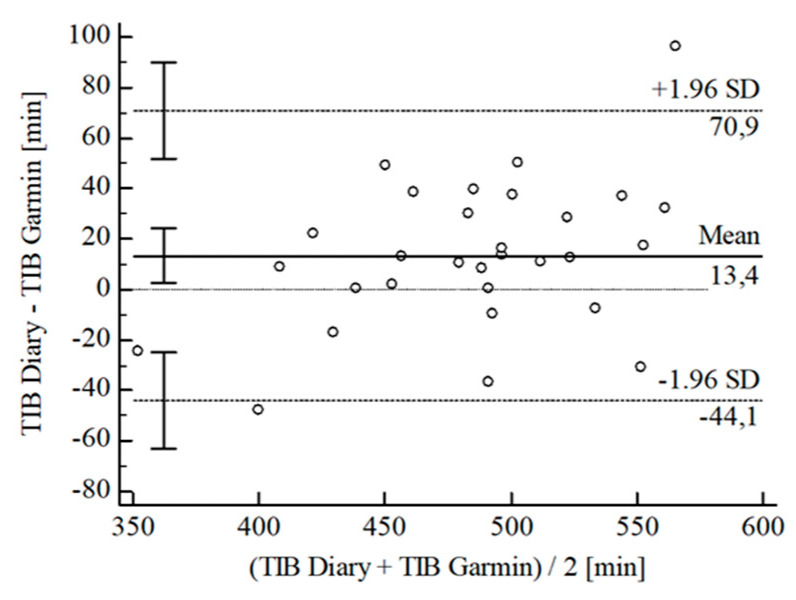
Bland-Altman plot for TIB Garmin^®^ vs. diary.

**Figure 5:**
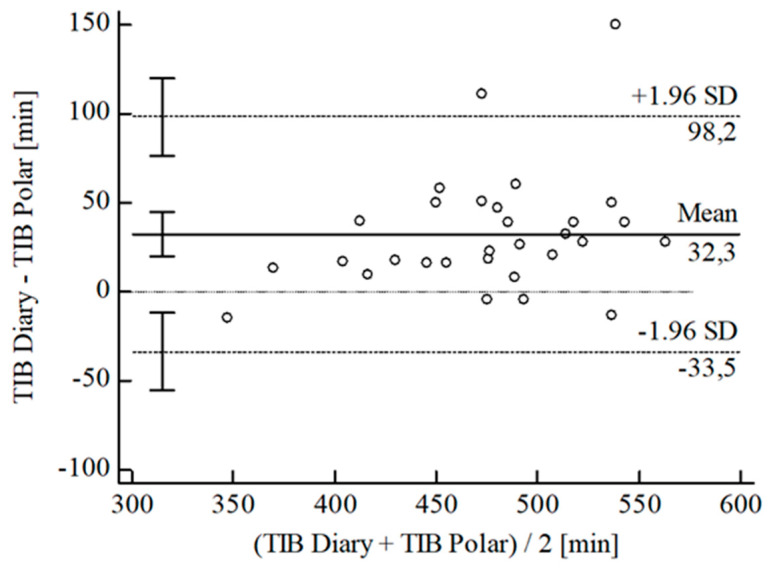
Bland-Altman plot for TIB Polar^®^ vs. diary.

**Figure 6:**
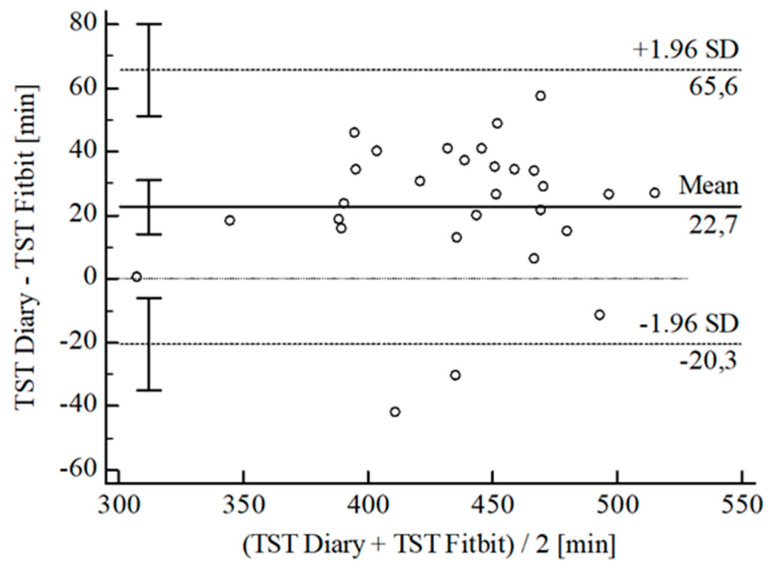
Bland-Altman plot for TST Fitbit^®^ vs. diary.

**Figure 7:**
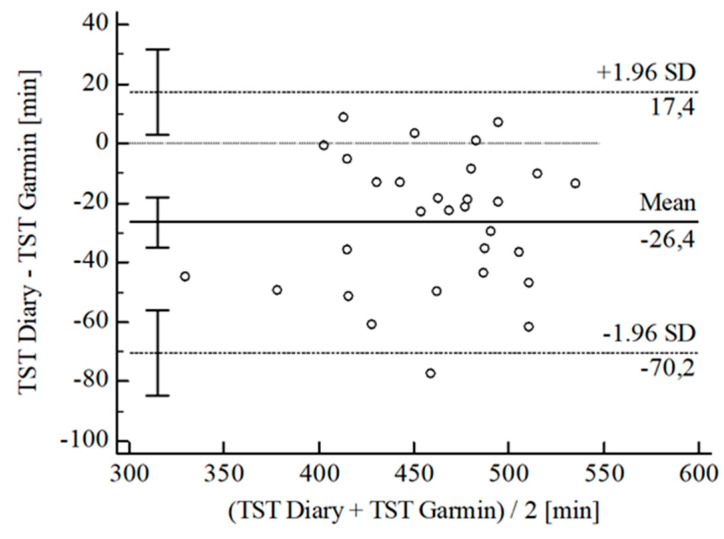
Bland-Altman Plot for TST Garmin^®^ vs. diary.

**Figure 8:**
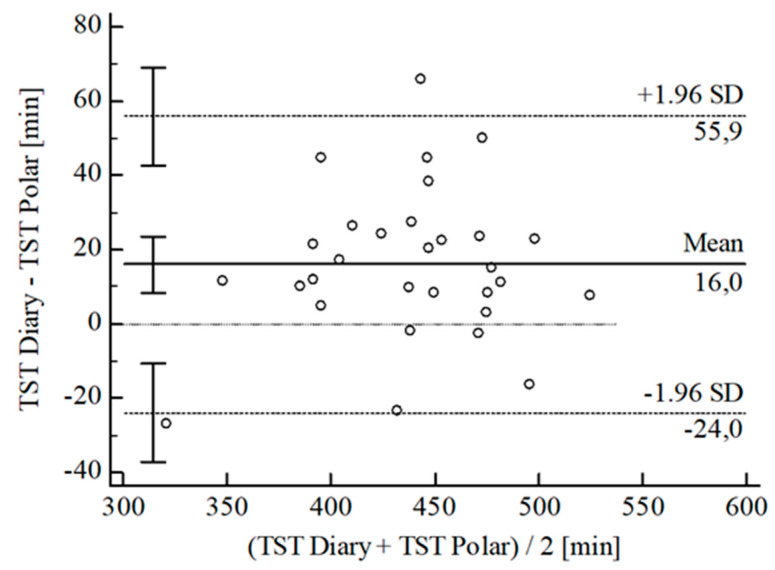
Bland-Altman plot for TST Polar^®^ vs. diary.

**Figure 9:**
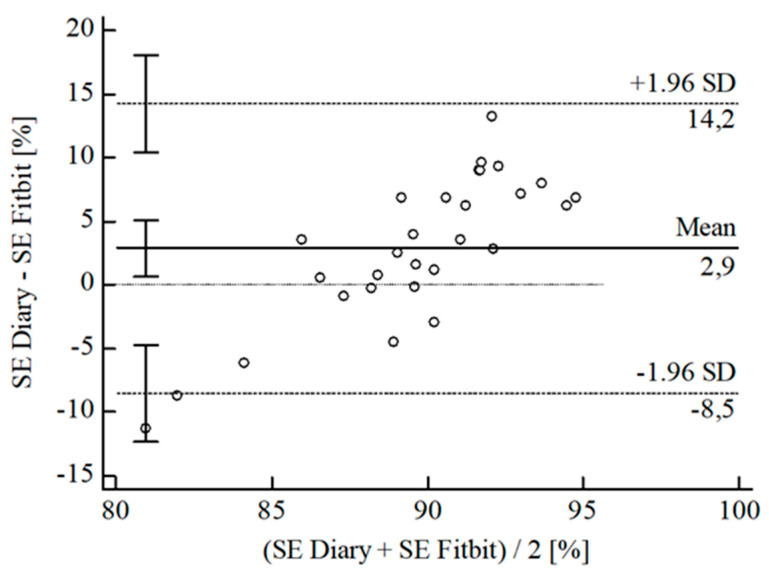
Bland-Altman plot for SE Fitbit^®^ vs. diary.

**Figure 10:**
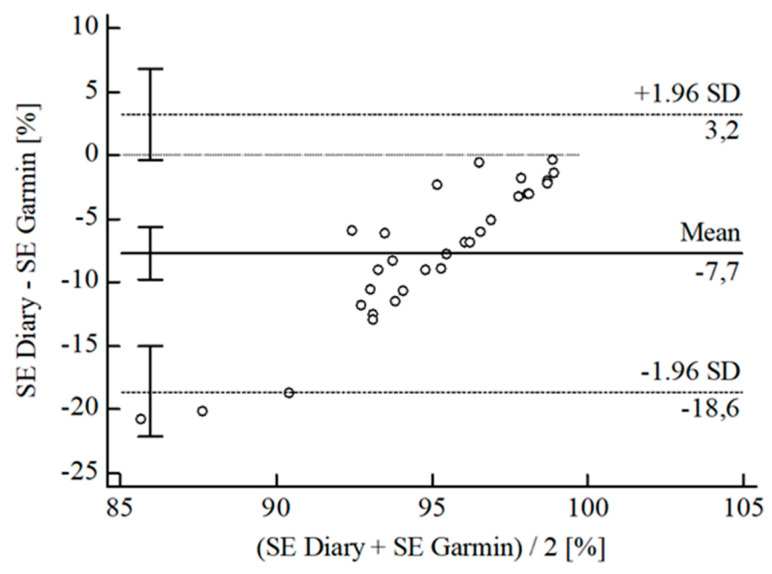
Bland-Altman plot for SE Garmin^®^ vs. diary.

**Figure 11:**
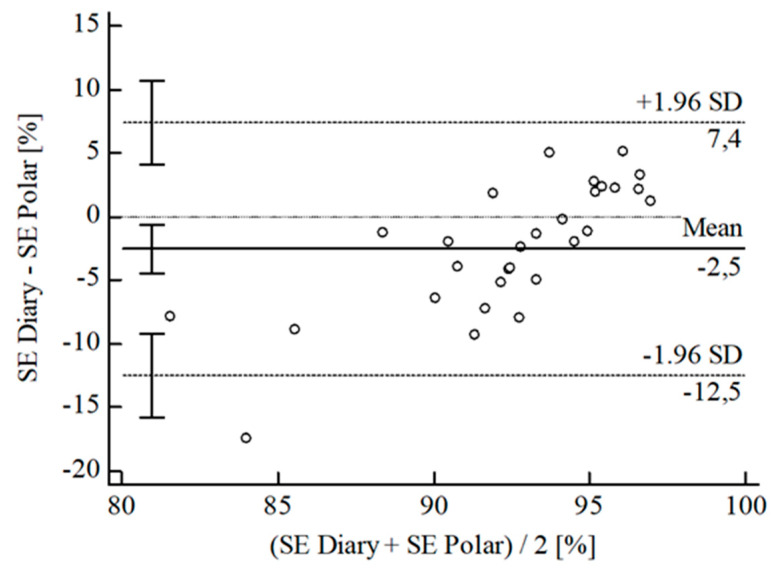
Bland-Altman plot for SE Polar^®^ vs. diary.

**Figure 12:**
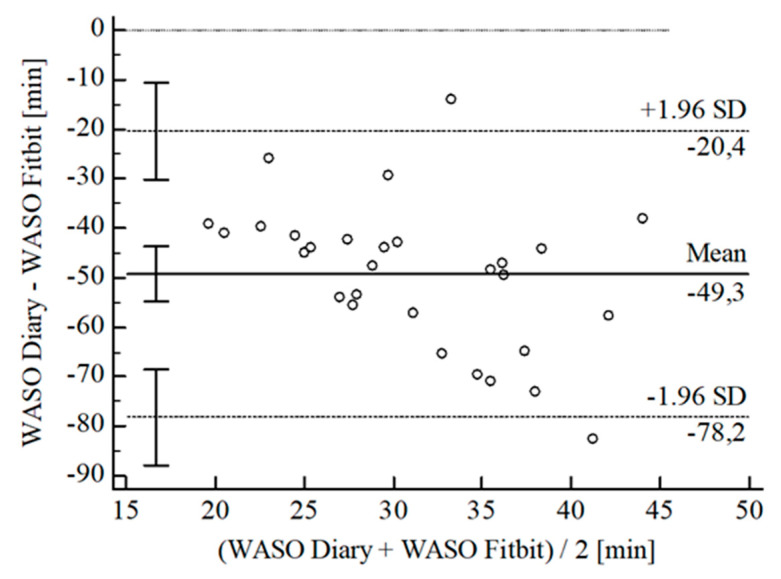
Bland-Altman Plot for WASO Fitbit^®^ vs. diary.

**Figure 13:**
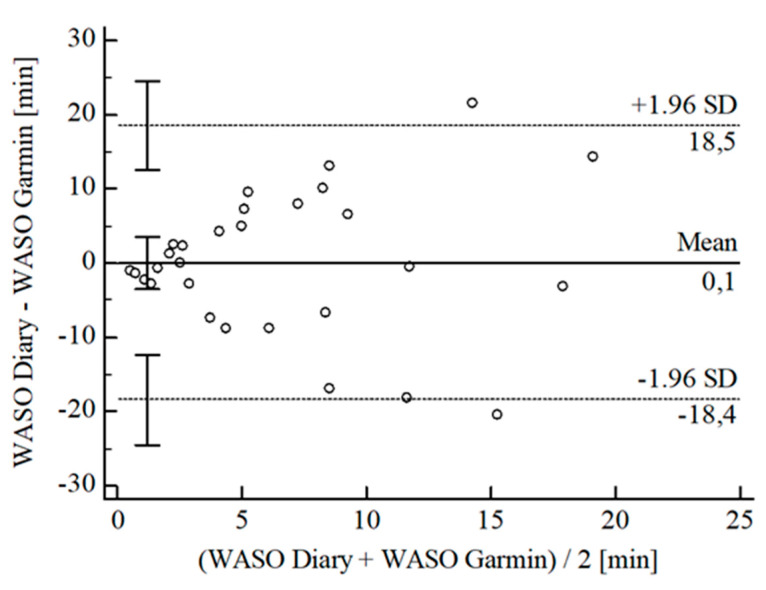
Bland-Altman Plot for WASO Garmin^®^ vs. diary.

**Figure 14:**
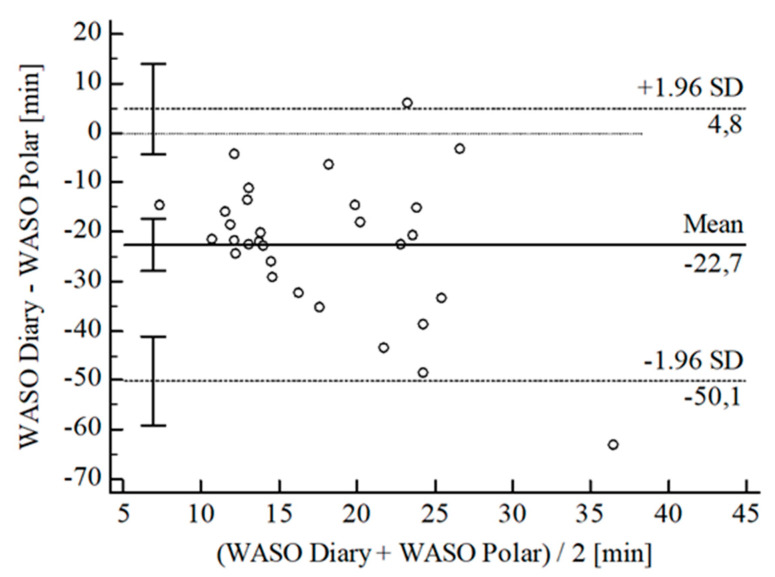
Bland-Altman Plot for WASO Polar^®^ vs. diary.

For the inferential statistical analysis at first, Bland-Altman plots were created for the variables TIB, TST, SE, and WASO to graphically illustrate the agreement between the devices and the sleep diary (see Figure 3, Figure 4, Figure 5, Figure 6, Figure 7, Figure 8, Figure 9, Figure 10, Figure 11, Figure 12, Figure 13 and Figure 14; for an overview of calculated values see Table A1 in Appendix A). The x-axis is the mean of both assessment tools, and the y-axis represents the diary minus the device with the line of equality (LoE) plotted at zero. Dotted lines are two standard deviations from the mean ($\bar{x}$ ± 1.96∙SD), and the highlighted sectors are the 95% confidence intervals (CI) of the mean and the limits of agreement (LoA). After removing outliers, and analyzing the reliability of the devices, we compared their measures with the sleep diary by calculating CCC_Lin_ as it allows us to quantify existing intraindividual concordance due to its comprising accuracy and precision subcomponent. If both measurement methods were completely in agreement, both the location and scale shift (accuracy) = 0, and the precision (correlation) r = 1, i.e., CCC_Lin_ = 1. Results were classified in addition to Cohen’s Kappa [40]. To calculate the mean differences between the devices and the sleep diary respectively, their percentage deviations, normality, and homoscedasticity of data must be given. Notably, no conclusions can be drawn about the sleep stages’ reliability because they have not been assessed by the sleep diary.

For TIB, the B-A-P of the Fitbit^®^ compared to the diary showed one measure outlying the 95% CI of the LoA (see Figure 3). After its removal, the computed agreement between the Fitbit^®^ and the diary was substantial (CCC_Lin_ = 0.75). As data were not normally distributed [W(28) = 0.86; *p* = 0.001], we performed a Johnson transformation [41]. Although transformed data again did not fit the assumption of normality [W(28) = 0.90; *p* = 0.010], the B-P-T revealed a homoscedastic distribution with χ^2^(1) = 0.02; *p* = 0.891. Hence, we calculated a mean difference of *Diary-Fitbit^®^* $\bar{x}$ = −0.15 units (SD = 0.53; 95% CI [−0.35; 0.06]. The LoE was within this 95% CI, and in general, 95% of the values were within a LoA-interval between −1.18 units (95% CI [−1.53; −0.82]; *Lower LoA*) and 0.89 units (95% CI [0.53; 1.24]; *Upper LoA*). The inverse transformation of data resulted in a mean difference of $\bar{x}$ = 0.86 units (SD = 1.70; 95% CI [0.70; 1.06] within a LoA-interval between 0.31 units (95% CI [0.22; 0.44]; *Lower LoA*) and 2.44 units (95% CI [1.70; 3.46]; *Upper LoA*), i.e., by chance, the Fitbit^®^ underestimated TIB on average by 5.33% (95% CI [2.82%; 7.84%]) with a range from −7.36% (95% CI [−11.71%; −3.02%]) overestimation to 18.02% (95% CI [13.67%; 22.36%]) underestimation of the subjective TIB. Including LoA-CI intervals, the maximal deviation was between 0.22 units (−11.71%) overestimation and 3.46 units (22.36%) underestimation.

The B-A-P comparing Garmin^®^ and the diary showed one measure outlying the 95% CI of the LoA (see Figure 4). After its removal, the computed agreement between the Garmin^®^ and the diary was almost perfect (CCC_Lin_ = 0.84). Data were normally distributed [W(29) = 0.96; *p* = 0.370], and the B-P-T showed a homoscedastic distribution with χ^2^(1) = 0.00; *p* = 0.968. The mean difference of TIB *Diary-Garmin^®^* was $\bar{x}$ = 10.54 min (SD = 25.24 min; 95% CI [0.93 min; 20.14 min]. The LoE was within the 95% CI, and the LoA-interval ranged from −38.95 min (95% CI [−55.55 min; −22.34 min]; *Lower LoA*) and 60.02 min (95% CI [43.41 min; 76.62 min]; *Upper LoA*), i.e., the Garmin^®^ underestimated TIB on average by 2.01% (95% CI [−0.05%; 4.08%]) with a range from −8.64% (95% CI [−12.21%; −5.06%]) overestimation to 12.66% (95% CI [9.09%; 16.23%]) underestimation of the subjective TIB. Including LoA-CI intervals, the maximal deviation was between −55.55 min (−12.21%) overestimation and 76.62 min (16.23%) underestimation.

The B-A-P comparing Polar^®^ and the diary showed one measure outlying the 95% CI of the LoA (see Figure 5). After its removal, the computed agreement between the Polar^®^ and the diary was substantial (CCC_Lin_ = 0.74), and S-W-T assumed normality [W(29) = 0.94; *p* = 0.077]. According to the B-P-T, the distribution was homoscedastic with χ^2^(1) = 0.01; *p* = 0.912. The mean difference of TIB *Diary-Polar^®^* was $\bar{x}$ = 28.30 min (SD = 25.70 min; 95% CI [18.52 min; 38.08 min]. The LoE was within the 95% CI, and the LoA-interval ranged from −22.08 min (95% CI [−38.98 min; −5.17 min]; *Lower LoA*) to 78.67 min (95% CI [61.77 min; 95.58 min]; *Upper LoA*), i.e., the Polar^®^ underestimated TIB on average by 5.90% (95% CI [3.83%; 7.97%]) with a range from −4.77% (95% CI [−8.35%; −1.19 %]) overestimation to 16.57% (95% CI [12.99%; 20.15%]) underestimation of the subjective TIB. Including LoA-CI intervals, the maximal deviation was between −38.98 min (−8.35%) overestimation and 95.58 min (20.15%) underestimation.

For TST, the B-A-P comparing the Fitbit^®^ and the diary showed one measure outlying the 95% CI of the LoA (see Figure 6). After its removal, the computed agreement between the Fitbit^®^ and the dairy was almost perfect (CCC_Lin_ = 0.83). Data were normally distributed [W(28) = 0.94; *p* = 0.108], and also the B-P-T revealed a homoscedastic distribution with χ^2^(1) = 0.16; *p* = 0.686. The mean difference of *Diary-Fitbit^®^* was $\bar{x}$ = 24.96 min (SD = 18.37 min; 95% CI [17.84 min; 32.09 min]. The LoE was within the 95% CI, and the LoA-interval ranged from −11.04 min (95% CI [−23.36 min; 1.28 min]; *Lower LoA*) to 60.96 min (95% CI [48.64 min; 73.28 min]; *Upper LoA*), i.e., the Fitbit^®^ underestimated TST on average by 5.72% (95% CI [4.11%; 7.35%]) with a range from −2.46% (95% CI [−5.26%; 0.34%]) overestimation to 13.91% (95% CI [11.11%; 16.71%]) underestimation of the subjective TST. Including LoA-CI intervals, the maximal deviation was between −23.36 min (−5.26%) overestimation and 73.28 min (16.71%) underestimation.

The B-A-P comparing the Garmin^®^ and the diary showed that all measures were within the 95% CI of the LoA (see Figure 7). The computed agreement between the Garmin^®^ and the diary was substantial (CCC_Lin_ = 0.76), and also the S-W-T showed a normal [W(30) = 0.97; *p* = 0.473] as well as the B-P-T a homoscedastic distribution [χ^2^(1) = 0.22; *p* = 0.637]. The mean difference of *Diary-Garmin^®^* was calculated with $\bar{x}$ = −26.40 min (SD = 22.36 min; 95% CI [−34.75 min; −18.05 min]. The LoE was within this 95% CI, and the LoA-interval ranged from −70.23 min (95% CI [−84.67 min; −55.80 min]; *Lower LoA*) and 17.43 min (95% CI [3.00 min; 31.87 min]; *Upper LoA*), i.e., by chance, the Garmin^®^ overestimated TST on average by −5.86% (95% CI [−7.78%; −3.94%]) with a range from −15.93% (95% CI [−19.24%; −12.61%]) overestimation to 4.20% (95% CI [0.89%; 7.52%]) underestimation of the subjective TST. Including LoA-CI intervals, the maximal deviation was between −84.67 min (−19.24%) overestimation and 31.87 min (7.52%) underestimation.

The B-A-P comparing Polar^®^ and the diary showed that all measures were within the 95% CI of the LoA (see Figure 8). The computed agreement between the Polar^®^ and the diary was almost perfect (CCC_Lin_ = 0.85). The S-W-T assumed normality [W(30) = 0.97; *p* = 0.485], and the B-P-T revealed a homoscedastic distribution of the measures with χ^2^(1) = 0.10; *p* = 0.756. The mean difference of TST *Diary-Polar^®^* was $\bar{x}$ = 15.95 min (SD = 20.38 min; 95% CI [8.34 min; 23.56 min]. The LoE was within 95% CI, and the LoA-interval ranged from −24.00 min (95% CI [−7.15 min; −10.84 min]; *Lower LoA*) to 55.90 min (95% CI [42.74 min; 69.05 min]; *Upper LoA*), i.e., the Polar^®^ underestimated TST on average by 3.59% (95% CI [1.80%; 5.38%]) with a range from −5.82% (95% CI [−8.92%; −2.72%]) overestimation to 13.00% (95% CI [9.90%; 16.10%]) underestimation of the subjective TST. Including LoA-CI intervals, the maximal deviation was between −37.15 min (−8.92%) overestimation and 69.05 min (16.10%) underestimation.

For SE, the B-A-P comparing Fitbit^®^ and the diary showed that all measures were within the 95% CI of the LoA (see Figure 9). Calculations resulted in a very weak agreement between the Polar^®^ and the diary (CCC_Lin_ = 0.12), although the S-W-T showed a normal [W(29) = 0.96; *p* = 0.327] and the B-P-T a homoscedastic distribution [χ^2^(1) = 0.49; *p* = 0.486]. The mean difference of *Diary-Fitbit^®^* was $\bar{x}$ = 2.87 min (SD = 5.79 min; 95% CI [0.67 min; 5.08 min]. The LoE was within the 95% CI, and the LoA-interval ranged from −8.47 min (95% CI [−12.28 min; −4.67 min]; *Lower LoA*) to 14.22 min (95% CI [10.41 min; 18.03 min]; *Upper LoA*), i.e., the Fitbit^®^ underestimated SE on average by 3.01% (95% CI [0.51%; 5.51%]) with a range from −9.87% (95% CI [−14.19%; −5.55%]) overestimation to 15.89% (95% CI [11.57%; 20.21%]) underestimation of the subjective SE. Including LoA-CI intervals, the maximal deviation was between −12.28 min (−14.19%) overestimation and 18.03 min (20.21%) underestimation.

The B-A-P comparing Garmin^®^ and the diary showed that all measures were within the 95% CI of the LoA (see Figure 10), but calculations resulted in no agreement between Garmin^®^ and the diary (CCC_Lin_ = 0.05). Thus, according to Landis and Koch [40], data interpretation of CCC_Lin_ ≤ 0.10 is not robust. We did not conduct further statistical analysis of SE Garmin^®^ vs. the diary.

The B-A-P comparing Polar^®^ and the diary showed one measure outlying the 95% CI of the LoA (see Figure 11). After its removal, the computed agreement between Polar^®^ and the diary was small (CCC_Lin_ = 0.34) whilst the S-W-T showed a normal [W(29) = 0.96; *p* = 0.240] and the B-P-T a homoscedastic distribution [χ^2^(1) = 0.98; *p* = 0.322]. The mean difference of SE *Diary-Polar^®^* was $\bar{x}$ = −2.03 min (SD = 4.30 min; 95% CI [−3.67 min; −0.40 min]. The LoE was within the 95% CI, and the LoA-interval ranged from −10.47 min (95% CI [−13.30 min; −7.64 min]; *Lower LoA*) to 6.40 min (95% CI [3.57 min; 9.23 min]; *Upper LoA*), i.e., Polar^®^ overestimated SE on average by −2.32% (95% CI [−4.12%; −0.51%]) with a range from −11.62% (95% CI [−14.74%; −8.50%]) overestimation to 6.98% (95% CI [3.86%; 10.10%]) underestimation of the subjective SE. Including LoA-CI intervals, the maximal deviation was between −13.30 min (−14.74%) overestimation and 9.23 min (10.10%) underestimation.

For WASO, the B-A-P comparing Fitbit^®^ and the diary showed that all measures were within the 95% CI of the LoA (see Figure 12). However, calculations resulted in no agreement between Fitbit^®^ and the diary (CCC_Lin_ = −0.01), for which reason we did not conduct further statistical analysis.

The B-A-P comparing Garmin^®^ and the diary showed that all measures were within the 95% CI of the LoA (see Figure 13). Again, calculations resulted in no agreement between Garmin^®^ and the diary (CCC_Lin_ = 0.10), and, referring to Landis and Koch [40], further statistical analysis would not be robust.

The B-A-P comparing Polar^®^ and the diary showed one measure outlying the 95% CI of the LoA (see Figure 14). After its removal, calculations resulted in no agreement between Polar^®^ and diary (CCC_Lin_ = −0.02). We did not conduct further statistical analysis.

## 4. Discussion

The aim of the present study was to assess the level of agreement of sleep measures between a sleep diary and three common consumer wearable devices. By comparing the wearables’ sleep measures with the subjective gold standard sleep diary, we wanted to test the devices as reliable tools for daily sleep assessment in young healthy adults in free-living conditions. Although sleep variables originate from the same objective sleep/wake experience of the individual, each assessment method depends on different source data to calculate these values. Whereas the wearables infer wake and sleep phases from changes in the amount of body movement and heart rate variability detected on the persons’ wrists, the sleep diary relies on the memory of the individual about their sleep/wake experience of the previous night. Thus, as each method can assess many of the same sleep/wake variables, it is reasonable to expect some agreement, but, at the same time, there might be substantial discrepancies due to the distinct source data.

For comprehensive proceeding, data collection was conducted over a period of four consecutive nights. Notably, wearables differed in all measurements: Despite zero missing rate, the Garmin^®^ device overestimated total sleep duration and was not able to detect sleep stages well. In contrast, the Fitbit^®^ device was more sensitive although wake times seemed considerably too long. At last, the Polar^®^ device did not only offer the most various parameters but also came closest to basic physiological sleep characteristics (e.g., 15–20% deep and REM sleep, 55–60% light sleep). In line with current literature, our results show that the wearables’ measures of TIB and TST can be considered reliable when compared with a sleep diary in healthy young adults in free-living conditions [30,42]. However, reliability might not be on hand regarding nightly interruptions and sleep stages. In particular, the latter occurs due to changes in brainwaves whereas wearables use heart rate recording and actigraphy for sleep assessment. The discrepancy in WASO might be due to the differences in sensitivity of the two assessment methods, i.e., individuals might be more likely to remember substantial awakenings whereas wearables might be more sensitive to detect also brief wake phases due to the actigraphic assessment of subtle wrist/body movements. Accordingly, we were able to replicate the findings of Jungquist et al. [43], Campanini et al. [44], and Thurman et al. [45].

Despite this limited data processing, in terms of consumer orientation, adequate knowledge and sleep tracking result from reliable sleep duration, approximate number and duration of awakenings after sleep onset, as well as the subjective feeling of recovery. Thus, for personal use, all three wearables depicted these data comprehensively. As the popularity of wearables is further booming and many people already use any kind of devices to track their daily life and share it with their friends and social community, additionally, usage in terms of health literacy, and therefore, adding the sleep component to one’s tracking habits, is highly feasible [13,46]. In line with this, the market changes more and more from health-related single-use to multifunctional gadgets [47].

To conclude, referring to Baron et al. [26] and Ibáñez et al. [48], the devices should primarily be used as an orientation of one’s own sleep behavior and as individual feedback on the individual sleep health status. In terms of health literacy, wearables seem a suitable tool to gather information about one’s sleep habits, and thereby, foster sleep health [49]. Especially during current pandemic times, the role of sleep for human well-being and functioning has become central. COVID-19 changed our lives and sleep. Whilst some do have more time to sleep, others suffer from sleep disturbances due to rumination and worries about the situation or the future [50]. Therefore, a proactive examination of their own sleep patterns and setting up healthy sleep and living conditions is crucial. Future research can start here by developing and empirically testing suitable sleep assessment and intervention tools.

According to the young consumer sleep technology research history, we want to emphasize our study’s strengths: First, it is one of the few and first investigations that took place in the field/under free-living conditions and not in the lab. Second, as it is known that the subjective rating of recovery is at least as important as good quantitative sleep values, we used a standardized sleep diary as a reference parameter for examining the wearables’ reliability. Third, we were able to track, in total, 120 nights consisting of four consecutive nights per person which is more than the recommended 72 h of tracking when using actigraphy [51]. Fourth, regarding the practical impact, we were able to show that the wearables, especially Garmin^®^ and Polar^®^, were in a reliable range compared to the sleep diary. Thus, it could be concluded that individuals can choose the wearable they prefer for tracking sleep and activity as they seem to be reliable tools, and as we found at least regarding sleep measures, none has the edge over the other.

Nevertheless, there are also some constraints limiting our study: First, we did not compare wearable data with the gold standard PSG, but focused on the quasi-gold standard sleep diary, i.e., correlating objective with subjective data. In addition, wearables do not measure sleep directly, and sleep parameters’ calculations are based on mathematical algorithms. This is why missing data or outlier values might occur in persons who toss and turn frequently during the night. Furthermore, due to the lacking access to raw data, consumers and researchers might not be able to notify when or if the wearables’ manufacturers change their algorithms. Second, participants got familiar with the handling of the devices in their preparation session and then tracked the investigated nights on their own responsibility. The same procedure was applied to the use of the sleep diary. Though, we assume that participants followed the described handling and study protocol as accurately as possible. Third, we conducted our study in free-living conditions and not in a laboratory. Whilst this setting made it possible for participants to keep their normal sleeping routine, a standardized approach including control of the side effects and possible disturbances could not be ensured in total.

## 5. Conclusions

In the current study, we found indications that wearables are reliable and highly feasible when compared to the subjective gold standard sleep diary. The greatest benefit of sleep tracking via wearables is the immediate increase in one’s sleep awareness. By wearing the device and dealing with the direct feedback, the sensitivity to their own sleep behavior increases. This, in turn, supports sleep health and positive (long-term) lifestyle changes, and at the same time, makes it possible to notice unhealthy sleep disturbances early. In sum, the use of consumer wearables seems to be a promising approach to assessing sleep in healthy populations in free-living conditions. Accordingly, future research should not only establish coherent study designs to investigate the usability as well as reliability and validity of wearable devices for sleep tracking in different target groups and settings but should also focus on the development of further monitoring devices using technological progress to facilitate their literal applicability in everyday life.

## Figures and Tables

**Table 1:** Technical specifications of the used wearables.

Device Name	Device Type	Measurement Strategy	PPG Technicalities	Battery Life
Fitbit Versa^®^ 2	Wrist-worn wearable	PPG Accelerometer	Contact PPG Red LED Reflective	4+ days
Garmin Fēnix^®^ 5X Plus	Wrist-worn wearable	PPG Accelerometer Gyroscope	Contact PPG Green LED Reflective	≤20 days
Polar Ignite^®^	Wrist-worn wearable	PPG Accelerometer	Contact PPG Green LED Reflective	≤5 days

PPG = photoplethysmography; LED = light emitting diode.

**Table 2:** Mean values of sleep variables.

	N	TIB (min)	TST (min)	SS (min)			SE (%)	WASO (min)
				REM	Deep	Light		
Fitbit Versa^®^ 2	29	463.1 ± 53.0	423.8 ± 45.6	89.9 ± 29.7	77.8 ± 18.0	254.6 ± 40.8	88.2 ± 1.9	55.9 ± 11.8
Garmin Fēnix^®^ 5X Plus	30	478.3 ± 47.0	472.6 ± 45.9	122.8 ± 43.2	65.9 ± 32.6	283.2 ± 35.9	98.7 ± 1.3	6.4 ± 6.8
Polar Ignite^®^	30	459.3 ± 48.8	430.2 ± 45.4	102.4 ± 18.8	78.0 ± 20.6	245.0 ± 37.8	93.6 ± 2.4	29.1 ± 11.3
Sleep Diary	30	491.6 ± 58.5	446.2 ± 47.7	not available			91.1 ± 5.9	6.4 ± 7.3

TIB = time in bed; TST = total sleep time; SS = sleep stages; REM = rapid eye movement; Deep = deep sleep; Light = light sleep; SE = sleep efficiency; WASO = wake after sleep onset.

## Data Availability

Not applicable.

## Appendix A

**Table A1:** Overview of agreement between wearables and diary (outliers removed).

		TIB			TST			SE			WASO		
		Fitbit^®^	Garmin^®^	Polar^®^	Fitbit^®^	Garmin^®^	Polar^®^	Fitbit^®^	Garmin^®^	Polar^®^	Fitbit^®^	Garmin^®^	Polar^®^
	N	28	29	29	28	30	30	29	30	29	29	30	29
**[min]**	$\bar{x}$ ± SD		10.54 ± 25.24	28.30 ± 25.70	24.96 ± 18.37	26.40 ± 22.36	15.95 ± 20.38	2.87 ± 5.79		2.03 ± 4.30			
	95% CI		0.93; 20.14	18.52; 38.08	17.84; 32.09	34.75; 18.05	8.34; 23.56	0.67; 5.08		3.67; 0.40			
	Upper LoA		60.02	78.67	60.96	17.43	55.90	14.22		6.40			
	95% CI		43.41; 76.62	61.77; 95.58	48.64; 73.28	3.00; 31.87	42.74; 69.05	10.41; 18.03		3.57; 9.23			
	Lower LoA		38.95	−22.08	11.04	70.23	24.00	8.47		10.47			
	95% CI		55.55; 22.34	38.98; 5.17	23.36; 1.28	84.67; 55.80	37.15; 10.84	12.28; 4.67		13.30; 7.64			
**[%]**	$\bar{x}$ ± SD	5.33 ± 6.47	2.01 ± 5.43	5.90 ± 5.44	5.72 ± 4.18	5.86 ± 5.13	3.59 ± 4.80	3.01 ± 6.57		2.32 ± 4.74			
	95% CI	2.82; 7.84	0.05; 4.08	3.83; 7.97	4.11; 7.35	7.78; 3.94	1.80; 5.38	0.51; 5.51		4.12; 0.51			
	Upper LoA	18.02	12.66	16.57	13.91	4.20	13.00	15.89		6.98			
	95% CI	13.67; 22.36	9.09; 16.23	12.99; 20.15	11.11; 16.71	0.89; 7.52	9.90; 16.10	11.57; 20.21		3.86; 10.10			
	Lower LoA	7.36	8.64	4.77	2.46	15.93	5.82	9.87		11.62			
	95% CI	11.71; 3.02	12.21; 5.06	8.35; 1.19	5.26; 0.34	19.24; 12.61	8.92; 2.72	14.19; 5.55		14.74; 8.50			
**over/underestimation**		↓	↓	↓	↓	↑	↓	↓		↑			
**CCC_Lin_**		0.75	0.84	0.74	0.83	0.76	0.85	0.12	0.05	0.34	0.01	0.10	0.02
**1 − ß**		1.00	1.00	1.00	1.00	1.00	1.00	0.10	0.06	0.48	0.05	0.09	0.05

Due to transformation TIB Fitbit^®^ cannot be given in minutes, and in general, agreement was only calculated if CCC_Lin_ was robust (> 0.10).
